# Fronto-striatal atrophy correlates of neuropsychiatric dysfunction in frontotemporal dementia (FTD) and Alzheimer's disease (AD)

**DOI:** 10.1590/S1980-57642013DN70100012

**Published:** 2013

**Authors:** Dong Seok Yi, Maxime Bertoux, Eneida Mioshi, John R. Hodges, Michael Hornberger

**Affiliations:** 1 Neuroscience Research Australia, Sydney, Australia.; 2 University Pierre and Marie Curie – Paris VI, Sorbonne Universités, Paris, France.; 3 Neuroscience Research Australia, Sydney, Australia. School of Medical Sciences, University of New South Wales, Sydney, Australia.; 4 Neuroscience Research Australia, Sydney, Australia. ARC Centre of Excellence in Cognition and its Disorders, Sydney, Australia. School of Medical Sciences, University of New South Wales, Sydney, Australia.

**Keywords:** frontotemporal dementia, Alzheimer's disease, neuropsychiatric symptoms, striatum

## Abstract

Behavioural disturbances in frontotemporal dementia (FTD) are thought to reflect mainly atrophy of cortical regions. Recent studies suggest that subcortical brain regions, in particular the striatum, are also significantly affected and this pathology might play a role in the generation of behavioural symptoms.

**OBJECTIVE:**

To investigate prefrontal cortical and striatal atrophy contributions to behavioural symptoms in FTD.

**METHODS:**

One hundred and eighty-two participants (87 FTD patients, 39 AD patients and 56 controls) were included. Behavioural profiles were established using the Cambridge Behavioural Inventory Revised (CBI-R) and Frontal System Behaviour Scale (FrSBe). Atrophy in prefrontal (VMPFC, DLPFC) and striatal (caudate, putamen) regions was established via a 5-point visual rating scale of the MRI scans. Behavioural scores were correlated with atrophy rating scores.

**RESULTS:**

Behavioural and atrophy ratings demonstrated that patients were significantly impaired compared to controls, with bvFTD being most severely affected. Behavioural-anatomical correlations revealed that VMPFC atrophy was closely related to abnormal behaviour and motivation disturbances. Stereotypical behaviours were associated with both VMPFC and striatal atrophy. By contrast, disturbance of eating was found to be related to striatal atrophy only.

**CONCLUSION:**

Frontal and striatal atrophy contributed to the behavioural disturbances seen in FTD, with some behaviours related to frontal, striatal or combined fronto-striatal pathology. Consideration of striatal contributions to the generation of behavioural disturbances should be taken into account when assessing patients with potential FTD.

## Abstract (pt)

Distúrbios de comportamento na demência frontotemporal (DFT) parecem refletir principalmente atrofia de regiões corticais. Estudos recentes sugerem que regiões cerebrais subcorticais, em particilar o estriado, são também são significativamente afetados e esta patologia pode ter um papel na geração dos sintomas comportamentais.

**OBJETIVO:**

Investigar a contribuição da atrofia cortical prefrontal e estriatal para os sintomas da DFT.

**MÉTODOS:**

182 participantes (87 pacienjtes com DFT, 39 pacientes com DA e 56 controles) foram incluídos. Os perfis cognitivos foram estabelecidos usando o *Cambridge Behavioural Inventory Revised* (CBI-R) e *Frontal System Behaviour Scale* (FrSBe). Atrofia nas regiões prefrontal (VMPFC, DLPFC) e estriatal (caudado e putamen) foi estabelecida através de uma escala visual de 5 pontos nas imagens de ressonância magnética. Os escores de comportamento foram correlacionados aos escores de atrfoia.

**RESULTADOS:**

Os resultados comportamentais e de atrofia demonstraram que os pacientes estavam significativamente mais comprometidos do que os controles, com os pacientes com DFT mais gravemente afetados. As correlações anátomo-comportamentais revelaram que a atrofia do VMPFC foi intimamente relacionada ao comportamento anormal e distúrbios de motivação. Comportamentos estereotipados estiveram associados com atrofia do VMPFC e estriatal. Em contraste, distúrbios da alimentação foram relacionados somente a atrofia estriatal.

**CONCLUSÃO:**

A atrofia frontal e estriatal contribuíram para os distúrbios vistos na DFT, com alguns comportamentos relacionados a patologia frontal, estriatal ou combinadas. Considerações quanto à contribuição estriatal na gênese dos distúrbios de comportamento devem ser levados em conta quando se avalia pacientes com DFT em potencial.

## INTRODUCTION

Frontotemporal dementia (FTD) can be divided into three clinical subtypes: behavioural frontotemporal dementia (bvFTD), semantic dementia (SD) and progressive non-fluent aphasia (PNFA). Patients with bvFTD, the most prevalent subtype, present with profound alteration in personality, social conduct and behaviour, which have been related to atrophy of prefrontal brain areas, particularly the ventromedial prefrontal cortex (VMPFC) but also anterior temporal atrophy.^1-4^ The clinical presentation of SD is characterized by anomia and impaired word comprehension^5^ with concomitant asymmetrical rostral temporal lobe atrophy.^6^ Finally, PNFA presents with expressive language deficits, characterized by effortful speech production, phonologic and grammatical errors,^7^ and atrophy in the left peri-Sylvian region.^8^ Although FTD is traditionally associated with cortical atrophy, which is thought to be the major determinant of their symptoms, there is growing evidence for concomitant involvement of subcortical brain regions that might be an important contributor to the symptoms. For example, a post-mortem study^9^ suggested that basal ganglia structures are affected from an early disease stage. At more advanced stages, basal ganglia degeneration was very evident as indicated by grossly dilated frontal horns of the lateral ventricles. These pathological findings were confirmed *in vivo*^10-13^ using MRI volumetrics demonstrating striatal atrophy especially in those with bvFTD and PNFA.^11,13,14^

These changes suggest a possible role of striatal atrophy in the genesis of symptoms in FTD. Previous studies investigating the relationship between striatal dysfunctions and FTD symptomatology have shown to correlation with disinhibition,^14,15^ functional disability^10^ and binge eating.^16^ Interestingly, the right side of the striatum has been mostly related to behavioural disturbances^13,14^ such as eating disorders,^15^ apathy,^17,18^ altered empathy,^19^ disinhibition^17^ and stereotypies,^20^ while the left striatum has been linked to cognitive impairments, such as language and executive dysfunctions.^13,21^

Overall, these findings suggest that behavioural symptoms may be associated with cortical and subcortical pathology in FTD. Surprisingly, to our knowledge, no study has investigated the effects of both prefrontal cortex and striatal atrophy on behavioural disturbances in FTD. Previous studies^4^ investigated whole brain correlates of behavioural disturbances, which were therefore exploratory. The current study aims to focus the contribution of the PFC and striatal atrophy in particular on behavioural disturbances in FTD. In addition, instead of using quantitative imaging methods as previously employed, we use a simple visual atrophy rating scale. Such ratings scales have the advantage that single patient coronal scans can be assessed in the clinic, without the need to employ sophisticated imaging analysis. Thus, establishment of the relation between symptoms and visual atrophy ratings, allows clinicians to corroborate the neural correlates of behavioural symptoms on the spot in the clinic for each patient. We predict that most behaviours would be correlated with prefrontal cortex atrophy, but that apathy and disinhibition might also show an association with striatal pathology.

## METHODS

**Participants.** One hundred and eighty-two participants were selected from the FRONTIER Clinical Database resulting in a sample of 44 bvFTD, 20 SD, 23 PNFA, 39 AD patients and 56 controls. All FTD patients met the current consensus diagnostic criteria for FTD^7,22^ while AD patients met the revised NINCDS-ADRDA diagnostic criteria for probable AD.^23^

Patient's disease severity was assessed with the Frontier Severity Rating Scale (FRS) scale.^24^ Rasch scores of the Frontotemporal dementia Rating Scale (FRS) (Mioshi REF) were used as markers of disease severity. The FRS is an assessment tool measuring change in everyday abilities (e.g. ability to use a telephone, taking correct medications, eating behaviours) and behavioural symptomatology (e.g., loss of affection and impulsivity). The FRS provides an index of dementia severity (very mild, mild, moderate, severe, very severe or profound) and is able to show differing rates of disease progression in FTD subtypes. A total raw score of 30 can be obtained on the FRS. Raw scores are first converted into percentage scores to respect a patient's premorbid abilities (e.g. no points are lost if the person has never cooked as part of his or her routine prior to disease onset). Percentage scores are then converted using a logit table into a logit score, which range from 5.39 (normal) to -6.66 (profound impairment). Logit scores aid in spreading the patients across the different severity categories and are based on a hierarchical analysis of item difficulty on the FRS.

Importantly, a drop of 1 logit score on the FRS therefore does not easily translate to either a change in the stage of dementia severity or a loss of 1 (out of 30) items on the FRS. Interpretation of a change in logit scores depends on the initial severity of dementia.

The reader is encouraged to obtain the conversion guide which is freely available from the Frontier Frontotemporal Dementia Research Group website (www.ftdrg.org).

All patients underwent a multidisciplinary assessment including clinical interview and examination by a senior neurologist (JRH), neuropsychological testing, structural MRI neuroimaging, as well as the carer's assessment of patient's behaviours. The study was approved by the University of New South Wales Human Research Ethics Advisory panel D (Biomedial, ref. #10035).

**Behavioural and cognitive assessment.** General cognition in all patients and controls was assessed via the the Addenbrooke's Cognitive Examination Revised (ACE-R).^25^ The ACE-R is a 100 point evaluation that assesses 5 cognitive domains: attention/orientation, memory, fluency, language and visuospatial.

Behavioural disturbances in the patients were assessed via: [1] Cambridge Behavioural Inventory Revised (CBI-R), which is an 81 item questionnaire that assesses cognitive, behavioural and affective symptoms as well as activities of daily living and evaluates various functional/behavioural domains using a 5 point rating scale.^26^ The following scores of the CBI-R were evaluated and analysed: abnormal behaviour, motivation, stereotypic and motor behaviour, mood, eating habits and beliefs; [2] Frontal System Behaviour Scale (FrSBe), which is a 46 item rating scale measuring apathy, disinhibition and dysexecutive functioning using a 5-point Likert scale completed by both patients and their carers.^27^

For both questionnaires, only data provided by the carer at the first clinic presentation was included in the analyses.

**Image acquisition & analysis.** All patients underwent the same imaging protocol with a whole-brain T1-weighted images using a 3-tesla Philips MRI scanner with standard quadrature head coil (coronal orientation, matrix 256x256, 200 slices, 1x1 mm^2^ in-plane resolution, slice thickness 1 mm, TE/TR=2.6/5.8 ms, flip angle α=19º).

**Figure 1:**
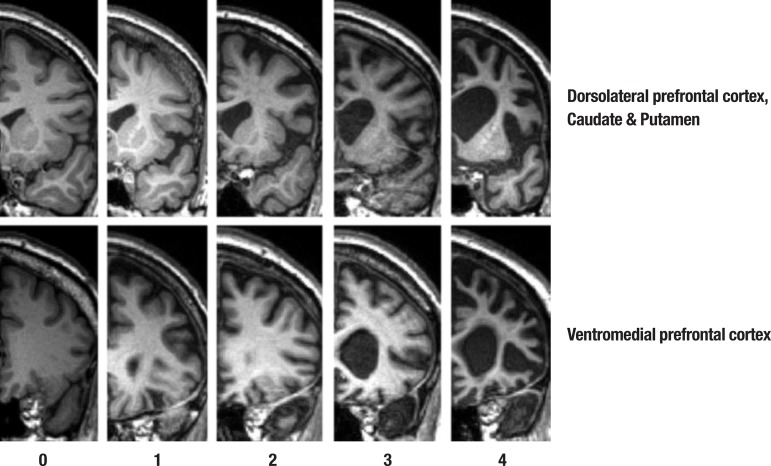
Shows the array of MR reference images and rating criteria employed in judging atrophy in the frontal lobe brain regions. Rating criteria range from 0=no atrophy to 4=severe atrophy for the rated brain regions.

One rater (DSY), blind to the clinical diagnosis, rated T1 coronal MRIs based on a visual rating scale developed by Davies and colleagues^28^ using a standard template against which to judge atrophy. The rater showed high reliability for the scoring of a MRI training set of 30 scans (Cronbach alpha=0.9). In brief, the rating method involved assessments of three coronal slices: the first at the level of the anterior temporal pole and the second at the level of the insula and third at the level of the posterior fornix. Firstly, two prefrontal regions were scored: ventromedial (VMPFC) and dorsolateral (DLPFC) prefrontal cortex. The total prefrontal cortex (PFC) atrophy was calculated by summing the scores of the two sub regions. The prefrontal rating method was identical to one of our previous studies.^29^ Based on our prefrontal rating method, we also developed visual ratings for striatal regions: caudate and putamen (Figure 1). Total striatum score was calculated by summing caudate and putamen atrophy scores. Atrophy within each region was rated on a 5-point Likert scale ranging from 0 to 4 (0=normal; 4=severe atrophy). The VMPFC was rated on the coronal image where the anterior pole is first visible. Dorsolateral frontal as well as caudate head and putamen regions were rated on the second coronal slice. This image was the most posterior slice through the temporal pole without visible connection between frontal and temporal lobes. The total prefrontal and striatal atrophy was obtained by averaging the atrophy ratings from the sub regions.

**Statistics.** Data were analysed using SPSS 18.0 (IBM Inc., USA). Parametric demographic (age, education, disease severity), neuropsychological (ACE-R), behavioural (CBI, FrSBe) and scan ratings (MRI) data were compared across the 5 groups (AD, bvFTD, SD, Control and PNFA) via one way ANOVAs followed by Tukey *post-hoc* tests. A priori, variables were plotted and checked for normality of distribution by Kolmogorov Smirnov tests. Variables revealing non-normal distributions were log transformed and appropriate log values were used in the analyses. Correlational analyses were corrected for multiple comparison via Bonferroni corrections.

## RESULTS

**Demographics and background.** Comparisons across the five groups (Table 1) revealed a significant difference in age (p<0.01) but not education or gender distribution (all p's>0.1). Post-hoc analysis showed that bvFTD patients and controls differed from each other (p<0.01) with bvFTD being significantly younger than controls. Age was therefore entered as a covariate in all remaining analyses.

**Table 1:** Average and standard deviations (SD) of demographics and cognitive tests

Demographics & cognitive tests	bvFTD	SD	PNFA	AD	Control	F value
N	44	20	23	39	56	
Age (years)	62.66 (8.95)^+^	64.84 (8.26)	67.84 (10.12)	64.28 (7.25)	68.46 (6.84)	*
Education (Years)	11.91 (3.13)	12.38 (3.59)	12.07 (3.76)	12 (3.14)	13.03 (2.80)	n.s.
Sex (M/F)	31/13	10/10	13/10	24/15	24/32	n.s.
ACE-R (100)	75.30 (14.82)^+^	54.05 (17.74)^+,++^	72.30 (17.26)^+^	68.64 (19.36)^+^	94.75 (3.61)	**
FRS Rasch logit score	-0.50 (2.02)	1.18 (1.55)	2.35 (1.70)	0.42 (1.07)	NA	**

n.s. not significant;

* p<0.01;

** p<0.001;

+ significantly different from controls;

++ significantly different from all other patient groups.

There were significant group effects for ACE-R and FRSRasch scores (p<0.001 for all). Follow-up post-hoc comparisons showed significantly different in ACE-R scores between the controls and patient groups (p< 0.001 for all). In addition, SD performed poorest across all groups (p<0.01for all) due to their pervasive semantic impairment.

**Behaviour.** For the CBI-R, there were main effects of group for all included scores (p<0.001for all). Not surprisingly, bvFTD patients were the most impaired for all behaviours showing significant effects compared to all other patient groups (p<0.05for all). The SD patients showed abnormal behaviour and stereotypical behaviour (p<0.05for all). The remaining patient groups (PNFA, AD) did not differ from controls or each other on any CBI score.

There were also significant group differences (p< 0.001 for all) for all categories of the FrSBe score. Similar to the CBI findings, bvFTD scored highest for all FrSBe scores (p<0.05for all) and SD patient were significantly impaired for the disinhibition subscore (p<0.05) compared to the remaining groups.

**MRI visual ratings.** Comparisons across prefrontal (VMPFC, DLPFC) and striatal (caudate, putamen) regions revealed significant group differences for all scores (p<0.001for all).

For the VMPFC, post-hoc tests showed that bvFTD was the only group with significant atrophy (p<0.05for all). By contrast, all patient groups showed DLPFC atrophy compared to controls (p<0.05) but they were not significant from each other (p>0.05for all).

For the striatal regions, post-hoc tests revealed caudate atrophy in all patient groups compared to controls (p<0.000for all). The bvFTD patients showed the highest atrophy ratings which were significantly higher than AD (p<0.01) and trending to significance compared to SD (p=0.065). A similar picture emerged for the putamen ratings, with bvFTD patients having the more significant putaminal atrophy compared to controls and AD (p<0.01for all) and marginal more significant atrophy compared to PNFA (p=0.067).

**Atrophy-behaviour correlations.** Abnormal behaviour and motivation dysfunction were correlated with VMPFC atrophy (r(180)=0.34; p<0.001; r(180)=0.41; p<0.001; respectively). By contrast, there were no associations between any of the behaviours measured and atrophy of the DLPFC, even when controlling for VMPFC atrophy. Interestingly, stereotypical behaviour was correlated with atrophy of the VMPFC, as well as the caudate and putamen (r(180)=0.39; p<0.001; r(180)=0.23; p<0.05; r(180)=0.25; p<0.05; respectively). The only behavioural disturbance that was exclusively associated with the striatum was disturbed eating, which correlated significantly with caudate and putamen atrophy (r(180)=0.26; p<0.025; r(180)=0.22; p<0.05; respectively).

To further tease apart the striatal and VMPFC contributions to stereotypical behaviours, we conducted partial correlations controlling for VMPFC atrophy. The results show that putaminal atrophy was still significantly correlated with stereotypical behaviour (r(180)=0.16; p<0.05;, whereas there was only a significant trend for the caudate (p=0.064).

## DISCUSSION

Our findings suggest that cortical and striatal pathology in FTD may have differential roles in the genesis of the behavioural symptoms which characterise FTD. While apathy was found to be strongly associated with VMPFC atrophy, stereotypical behaviours were related to both cortical and striatal pathology (caudate and putamen) and eating disturbance was exclusively associated with basal ganglia changes. Interestingly, these findings did not fulfil our predictions completely. Although most behavioural symptoms were associated with PFC atrophy, apathy and disinhibition did not show striatal atrophy correlates.

In keeping with prior studies, patients with bvFTD showed the highest rate of all behavioural disturbances^30,31^ although those with SD also demonstrated stereotypical behaviours.^32,33^ The bvFTD group had the greatest atrophy ratings for all prefrontal cortex regions (VMPFC and DLPFC), with particular involvement of the former region compared to all other groups.^29,34^ By contrast, the degree of involvement of the DLPFC was equal across dementia groups including AD which suggests that DLPFC atrophy has little diagnostic utility. Moreover, patients with all three FTD subtypes as well as AD showed atrophy of the striatum with a graduation in that those with bvFTD showed the most severe involvement followed by PNFA and SD. These findings confirms most previous results,^9-11,14^ although striatal atrophy has not been a universal finding in SD.^13,35^

Stereotypical behaviours were related to both striatal and prefrontal atrophy ratings which indicate that pathology in either region may be important in the pathogenesis of these behaviours. Josephs et al.^20^ in a pathology-based study also showed that frontal and striatal atrophy are were associated with stereotypies in FTD. Stereotypical behaviours are, however, complex, ranging from simple motor stereotypies, such as clapping, foot-tapping, and utterances, to more complex behaviours, including wandering or rearranging objects. It is currently not clear whether all these behavioural changes can be attributed to a common neural origin. Future investigations should elucidate whether simple versus complex stereotypies rely on the same neural regions and the specific roles of prefrontal vs. striatal regions.

In contrast to stereotypical behaviours, motivational dysfunction and abnormal behaviours were related to VMPFC atrophy. Previous studies have also implicated the VMPFC in disturbances of motivation^17^ although the exact role of this region in the complex processes that govern goal setting; goal achievement and reward remain unclear. The VMPFC also plays a crucial role in the maintenance of normal social and emotional behaviour^36^ a full discussion of which is beyond the scope of this paper (for review see^37^).

Finally, changes in eating, which tend to manifest as increased appetite with reduced satiety and a craving for sweet food, was correlated with striatal rather than cortical atrophy. Eating disturbances have been previously found to be associated with subcortical dysfunctions in FTD, notably changes in satiety that have been linked to hypothalamic pathology in FTD^38^ and binge eating, which has been related to striatal and cortical atrophy,^16^ It is possible therefore that the correlation in our study was driven more by stereotypic changes in feeding. The exact role of striatal versus other subcortical structures is clearly worthy of further investigation.

In summary, our findings show that the striatum is extensively involved in FTD symptomatology, particularly in the behavioural disturbances that typify this disabling disorder. The interaction of prefrontal and striatal regions is crucial in the maintenance of normal behaviour. It is advisable to take pathology of subcortical regions into account when assessing patients with potential FTD, and bvFTD in particular. Our findings require confirmation and elucidation using quantitative MRI methods combined with more detailed evaluation of aspects of symptoms such as the stereotypic and eating behaviours. Further studies should as well investigate the differential involvement of left/right striatal structures in both behavioural and cognitive disturbances.

## Figures and Tables

**Figure 2:**
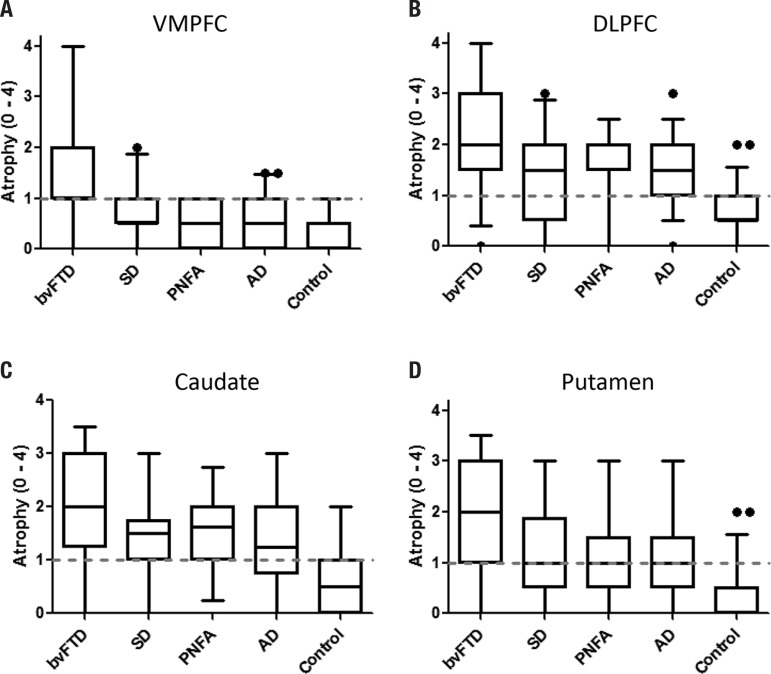
Shows boxplots for atrophy ratings in [A] VMPFC, [B] DLPFC, [C] caudate and [D] putamen brain regions across all participant groups. The dotted line indicates the threshold from which on a rating is considered to be definite atrophic. Boxplot whiskers indicate 5-95% confidence intervals.

**Figure 3:**
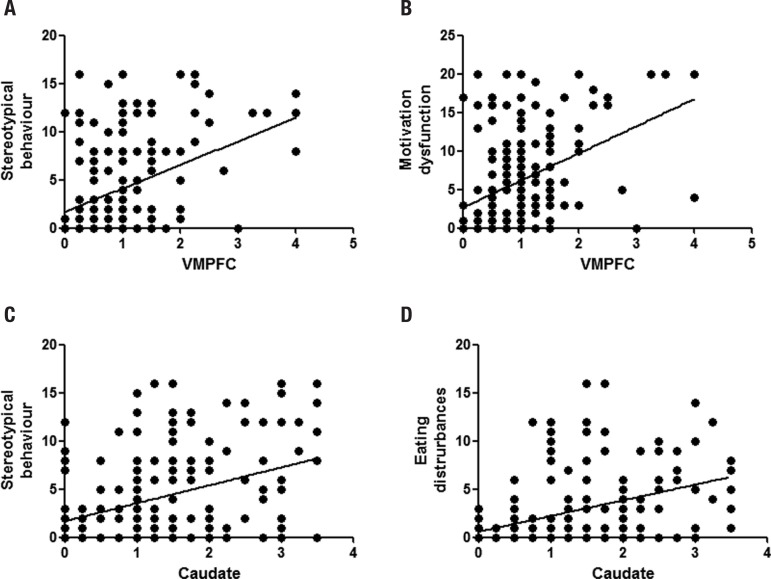
Shows the correlation of [A] VMPFC atrophy ratings with stereotypical behaviour, [B] VMPFC atrophy rating with motivational disturbance, [C] caudate atrophy ratings with stereotypical behaviour and [D] caudate atrophy rating with eating disturbances. The line indicates the best-fit linear regression slope for each graph.

**Table 2:** Averages and standard deviations (SD) of the behavioural questionnaires.

Behaviour		bvFTD	SD	PNFA	AD	Control	F value
**CBI**	Abnormal behaviour	9.60 (6)**^,+,++^	4.80 (5)**	1.78 (4)	2.92 (4)	0.74 (2)	*
	Motivation	12.88 (6)**^,+,++^	6.75 (6)**	5.04 (4)**	6.66 (6)**	0.48 (1)	*
	Stereotypical behaviour	8.57 (5)**^,+,++^	6.50 (6)**^,+,++^	2.35 (3)	3.34 (4)**	1.04 (2)	*
	Mood	5.21 (3)**^,+,++^	3.40 (3)**	2.52 (3)**	3.34 (3)**	0.43 (1.95)	*
	Eating	7.00 (4)**^,+,++,§^	2.45 (3)	0.87 (2)	2.24 (3)	0.66 (1)	*
	Beliefs	1.22 (2)**^,++^	0.50 (1)	0.04 (0)	0.74 (2)	0.00 (0.00)	*
**FrSBe**	Apathy	87.00 (22)^+,++^	79.50 (21)	63.18 (16)	67.86 (17)	-	*
	Disinhibition	75.59 (20)^+,++^	75.41 (28)^+,++^	51.82 (12)	56.46 (16)	-	*
	Executive	81.03 (20)^+,++^	75.65 (20)	60.41 (14)	69.29 (14)	-	*

* p<0.001;

** significantly different from controls;

+ significantly different from AD;

++ significantly different from PNFA;

§ significantly different from SD.
